# The Nursing Supplement

**Published:** 1888-04-21

**Authors:** 


### The Nursing Mirror.

### En Passant.

LEWES INFIRMARY.—The present matron of the Lewes Infirmary is retiring, after 21 years of service, on account of ill-health. It is not often that a matron remains so long at one post, but as there is at present only accommodation for five patients in the infirmary, the duties cannot be arduous. When the new wing is finished, which is being built as a Jubilee commemoration, there will be room for about seventeen beds, and rumour suggests that a female ward may be added in memory of the late Mayor of Lewes.

ANNUAL MEETINGS.—There have been several large meetings in connection with various hospitals lately, and at many of them a word of praise was reserved for the nursing staff. The Lord Mayor, in proposing prosperity to the German Hospital, said he specially wished to pay a warm tribute of respect to the staff of sisters—ladies of education and good station in society—for devoting their lives to the relief of the suffering patients. At the meeting in connexion with the London Hospital it was noteworthy that a Roman Catholic bishop and the Chief Rabbi both spoke gratefully of the attention given to patients of all denominations in our largest hospital. The Jews were enabled to keep the Passover as perfectly in the Hebrew wards as they could do in their own homes.

VOLUNTEER NURSES.—Miss Florence Nightingale, writing to the promoters of a bazaar in aid of the funds of the Manchester Volunteer Medical Staff Corps, explained that she was unable to accept invitation to be present, being a permanent invalid, and almost entirely a prisoner in her own room. With regard to the proposed nursing sisters, Miss Nightingale wrote: "If really trained and with some experience, they will be a valuable addition should ever the Volunteers have to take the field."

LADY DOCTORS.—Miss Macdonald has passed the recognised course of a medical school and has been admitted by the Society of Apothecaries to examination for its diploma in medicine, surgery, and midwifery. The ice having been thus successfully broken, the society will henceforth admit any lady to its examination, who can fulfil the same conditions. Bravo! Miss Macdonald.

NURSING IN CANADA.—Many nurses having applied to us for information as to the prospects of a nurse who emigrates to Canada, we have made inquiries from the Canadian Government, and can hear of no institution which would promise them work on their arrival. No doubt a few well-trained nurses would always be able to get employment, but we should advise only those who have friends on the other side to risk the probability.

LADY NURSES IN INDIA.—The *Times of India* thus records the arrival of Miss Loch and her staff of nurses: "Ten lady nurses have arrived in the troopship *Malabar* on the 21st of March. One lady superintendent and five nursing sisters are for duty at the Military Station Hospital at Rawal Pindi, and one lady superintendent and three nursing sisters for Bangalore. The pay of the lady superintendent is Rs. 400 per mensem, and of the nursing sisters Rs. 175 per mensem. The employment of nursing sisters in military hospitals is, we think, a move in the right direction, and we have no doubt that their services will be duly appreciated by the soldiers. The ladies are chiefly from St. Bartholomew's and Guy's Hospitals, London. We would like to see some being sent for the Bombay Presidency. Much useful work could be found for them at Poona, Mhow, and Bombay." From £360 to £480 a year is a good salary for a lady superintendent, and wages of from £160 to £210 for sisters is good pay, and contrasts favourably with the salaries given to the heads of nursing departments of hospitals and institutions in this country.

THE "NURSING RECORD."—The statements made in this paper concerning the Pension Fund and the various branches of The Hospitals Association's work are so evidently written in a hostile spirit that we need scarcely warn our readers not to accept them as unbiassed facts. In such circumstances we regret to hear that this paper is regarded as representing the views of the leading spirits and active promoters of the British Nurses' Association, and their names have been attached to several of the articles. Yet we can hardly credit the rumour, seeing that the declared object of the British Nurses' Association is to raise nursing to the level of a profession. If this be its object, how can the promoters allow their paper to join the *Lancet* in its attemp to deprive nurses of the munificent co-operation of the merchant princes by wrecking the National Pension Fund for Nurses? Nurses are no doubt too sensible to be misled by statements so evidently unreliable and interested. It is, however, well that all should take note of the fact that the first act of the reputed organ of the British Nurses' Association, if successful, will deprive nurses of the security and comfort of a permanent provision in the day of their necessity. If these opponents of the Pension Fund were to succeed they would leave nurses absolutely dependent upon the casual charity of strangers. What a fall is here, and what confidence can nurses put in such would-be leaders as the promoters of the British Nurses' Association thus show themselves to be? The actuary, Mr. George King, is responsible for the figures in the tables, and we advise every nurse to read what he says on the next page.

THE NATIONAL PENSION FUND.—A CAUTION.—Certain persons and newspapers which may be supposed to have some influence with a portion of the nursing profession are making persistent efforts to discredit the Pension Fund, accusing the actuary of making the tables of payments too high. The hollow and unworthy character of such criticisms is proved by the fact that the chief of these journals refuses to publish the actuary's reply, exposing the falseness of its statements, charges, and figures. It is necessary to point out, therefore, that nurses and other hospital officials will do well to remember that those who can really decide the points at issue are persons accustomed to figures—hospital secretaries, for example, or, better still, actuaries and insurance managers. The details of the Fund have been worked out entirely by actuaries of the highest eminence, and they alone are responsible for the rates of premium and the amounts of pension. The largest possible pensions are given in every case which the premiums paid will produce, compatibly with safety. The Fund must be absolutely safe. It is, as a matter of fact, as safe as Government annuities or the Bank of England. The points to remember are these: that it is certain that no other public body or fund can safely give better terms than this Fund; and that in addition to the pensions to which nurses will be entitled by their own payments, the Fund has behind it, in addition to the Bonus Fund, the interest of £26,000, or about a thousand a-year, to be distributed equally among the holders of pensions, for which they pay nothing at all. Nurses will therefore be well advised to give no heed to reckless statements made by persons who, apparently, would rather that nurses should have no pensions at all, than that they should have them from any source not controlled by themselves.

### The National Pension Fund for Nurses.

### LETTERS FROM THE ACTUARY.

In the *Lancet* of the 7th inst. there was an article on the National Pension Fund for Nurses which was full of misstatements and misrepresentations. I sent a reply, but the editor refused me a hearing, preferring to insert a second article, if possible more misleading than the first. The matter is of great public importance, and the articles, if uncontradicted, will do harm. I beg of you, therefore, in the cause of justice, to give publicity to my letter to the *Lancet*, of which I enclose a copy.

George King, Actuary.

92, Cheapside, E.C., April 18th, 1888.

"*To the Editor of the* '*Lancet.*'

"Sir,—On page 684 of to-day's *Lancet* there is an article on 'The National Pension Fund for Nurses,' in which you profess to show that 'the promoters of the society have fallen far behind the open market,' and in which you declare that 'it is a very bold thing to ask a policyholder to speculate on a vague promise,' 'to the extent of adding 37 per cent. to her annual premium.'*

"In times past the *Lancet* has been looked upon as the leading medical journal, and any statements made in its columns are received with credence. I think it therefore due to myself, as the actuary responsible for the tables and regulations of the National Pension Fund, to request further particulars in support of your statements. I am entitled to ask you to give the names of the 'great insurance companies' which charge for the same benefit rates 37 per cent. lower than those I have advised the Council of the National Pension Fund to adopt.

"According to the published prospectus of the fund, the annual contribution from a nurse aged 20 for a pension of £15, to commence at 50, is £4 11*s.* 8*d.*, as pointed out by you. Government, for precisely the same benefit, charges £4 18*s.* 9*d.* Thus, it appears that, so far from being behind the open market, I have seen my way to advise the National Pension Fund to accept rates more than 7 per cent. lower than those required by Government. Moreover, the annuity offered by Government is a *maximum* benefit, while that offered by the National Pension Fund is a *minimum* benefit, which will certainly be increased by bonuses. There already exists a bonus fund of £1,000 per annum, and this, to my knowledge, could have been doubled; but the further gifts offered have for the present been discouraged, because the object of the promoters of the fund is to help the nurses to help themselves, and not at all to destroy their self-reliance and self-respect by doling out to them charitable relief.

"On personal grounds I have asked you to substantiate your statements, but I think the duty is incumbent upon you in the interests of the public also.

"If some of the great insurance companies are doing annuity business at rates 37 per cent. below those of the National Pension Fund, then, according to the figures quoted above, their rates must be 44 per cent. below those of the Government. Now, the great majority of insurance companies have long ceased to transact annuity business, because they cannot profitably compete with Government, and those few which have continued to grant annuities have been losing money over them. A company, therefore, which abates 44 per cent. on Government prices deserves to be exposed, and a prudent nurse would not care to invest her savings in such an institution. Dare you advise her to do so? She will do much better by joining the National Pension Fund. There, it is true, her contribution will be only 7 per cent. less than that charged by Government, but her minimum pension will be absolutely safe, and the already large bonus fund guarantees that it will be considerably increased.

"In my opinion journalists and others who try to render the National Pension Fund abortive incur a very grave responsibility. Such an opportunity will never recur of assisting a hard-working body of public servants towards self-help and independence.

"I should like to add a word regarding the regulations of the fund on which you animadvert, but I have already occupied too much of your space. There is nothing oppressive in them, as you seem to suppose. They are only those that the experience of the best friendly societies shows to be necessary. The enclosed copy of my report* to the promoters gives explanations in more detail, and will, I think, make this point, as well as others, clear to you.

"I remain, your obedient servant,

"(Signed) George King, Actuary.

"92, Cheapside, London, April 7th, 1888."

* Since this letter was written the *Lancet* has confessed to a mistake, and reduced the figure to 27 per cent., but the change does not affect my argument.—G. K.

* [We hope to publish this report next week.—Ed. *T. H.*]

### APPLICANTS' DIFFICULTIES.

Many nurses have begged me to ask you to kindly insert the following questions, which have been put by many applicants for the Nurses' Pension Fund, with the answers attached, as it might tend to simplify matters. Also kindly make known to the nurses that it would save much trouble if they would insert the number of the table in the prospectus under which they join the Fund.

Philip Grove, Secretary.

National Pension Fund, 38, Old Jewry, E.C.

Questions asked by Applicants and Answers Sent.

| | |
|---|---|
| 1. Can a nurse make a retrograde movement? *i.e.*, can a nurse of (say) 40 enter as of (say) 38 years by paying up arrears by a lump sum? | 1. Yes; any possible variation can be no doubt arranged for. Nurses should at once send a clear statement of what they want to the Secretary. |
| 2. Is it necessary that pensions should actually commence at 50, 55, or 60, in the event of their not being required, as it would give a longer time in which to be returnable? | 2. A nurse entitled to a pension at a given age can, (1) when the time arrives, re-invest the pension in the Fund to produce larger benefits at a later period, or (2) enter for pension to commence at other ages than those stated. |
| 3. What length of notice is required to withdraw contributions? | 3. This must be settled by the Council. The notice will certainly not be less than the intervals of payment. Thus, a quarterly contributor would probably have to give a quarter's notice, subject to the discretion of the Council in particular cases. |
| 4. Table V.—Are contributions returnable on death or withdrawal, as in other tables, or only on death (if before age 50)? | 4. Refer to table and you will find two columns, one returnable the other not. |
| 5. Do contributions commence when applications are approved, or do they wait until 1,000 join? | 5. It has already been stated that applications must come before the Council, and when approved notice will be sent to the applicants. It is probable that the lawyers will hold that 1,000 must first apply, so everybody interested should canvass their friends. |
| 6. Table VI.—Are there bonus additions? | 6. Certainly. |
| 7. Are dispensers eligible as *Hospital Officials* for pension? | 7. Certainly. |
| 8. Can a nurse enter in more than one table at the same time? | 8. Certainly. (See answer to Question 1.) |
| 9. If a nurse out of employment or by any chance could not meet a quarter's payment, could it be paid up at a later date, or would the pension fall through? | 9. Arrangements can be made to meet all such cases, but they must, of course, vary in each instance according to circumstances. |
| 10. Would a nurse temporarily leaving the United Kingdom be entitled to sick pay? | 10. Yes. |

### The Directory for Nurses, Philadelphia.

We have received an account of a Directory for Nurses at Philadelphia, worked on a different plan from any existing in this country. It appears to be a useful and successful scheme, and in laying it before our readers we should be glad if they would give it their consideration, and we invite correspondence anent it.

The Directory is in connection with the College of Physicians, and has been in existence about four years. It supplies information regarding male and female nurses to the public, at a fee ranging from one to ten dollars, according as the information leads to an eegagemont or not. A nurse upon applying to be registered must produce testimonials or certificates from a training school; also from any physicians or families for whom she has nursed. If these are satisfactory her name is entered on the register of the Directory on payment of a fee of five dollars (one guinea). Nurses are registered to receive from ten to thirty dollars a week, which is a much higher rate of payment than prevails in England. The nurse is then subject to the following rules :—

I. The commencement, and also the termination of every engagement, whether obtained privately or through the Directory, must be reported in writing by the next mail. Verbal reports will not be accepted.

II. Change of residence, sudden illness, temporary absence from home, or any other cause which will prevent the prompt acceptance of an engagement, must also be reported by the next mail.

III. Nurses may charge less, but not more, than the rates they have registered. They may, however, change their registered rates whenever they please.

IV. An engagement tendered through the Directory must be accepted if the case be such as the nurse is registered for, and the registered price offered. Illness so sudden as not yet to have been reported to the Directory is the only valid excuse.

V. The first failure to comply with any of the foregoing rules will be followed by a printed warning; the second, by the removal of the nurse's name from the register, in which case it can be restored only at the discretion of the Committee, and by a second payment of the registration fee of five dollars.

VI. The fees for telegrams, messenger boys, etc., must be paid by the nurse who obtains the engagement.

VII. The Committee reserve the right to remove the name of any nurse from the register, for what to them may seem sufficient cause.

VIII. When once an engagement has been definitely made, it must not be broken, save by mutual agreement.

To facilitate the reports required by rules I. and II., addressed postal cards with printed blank forms are sold by the Directory at cost price. The foregoing rules are intended to promote the welfare and the interests of the nurses as well as those of the Directory. The value of the Directory to the public depends chiefly upon the quickness with which it can meet the demands made upon it, and this can be secured only by the accuracy and promptness of its information.

The two chief points in this scheme are that only certificated nurses are registered, and that the nurses receive their own earnings. The question of registration is one to which the attention of nurses has been largely directed of late, and one which requires to be looked at from many points of view. Some of us fear that with legal registration we might bind about our necks a grievous burden, and put ourselves in the power of others to a larger extent than would be comfortable. Present private institutions are severely feeling the competition of the newly-started homes in connexion with the hospitals for sending out private nurses. As time goes on and the present medical students go forth to practice, they are sure to send to their own hospitals for nurses, and gradually some of the private institutions will have to give way. Will they then form themselves into a Directory, which gives the nurses a maximum of freedom while securing their absolute efficiency ? The whole subject is fraught with difficulties which time will doubtless dissolve, and in the meanwhile the best plan seems to be in no hurry to accept the invitation to walk into anyone's parlour, but to listen to all sides and discuss the question with those who have any knowledge of it, whenever it is possible.

### Everybody's Opinion.

### THE HOLLOND INSTITUTION, NICE.

The Nurses of the Hollond Institution will feel much obliged to the Editor of The Hospital if he will publish the enclosed letter in the "Nursing Mirror" as soon as possible. "The present nurses of the Hollond Institution at Nice wish to express their thanks to 'F. M., at Birmingham,' for having kindly taken up the cudgels on behalf of their directress before they had had time to do so themselves. They also wish to state, for general information, that, owing to her extreme kindness and good management, the institution at Nice is a real 'home' to them, and not one only in name."

[It gives us much pleasure to do this act of justice, and we congratulate the writers on their loyalty.—Ed. *T. H.*]

### FREE TRAINING IN MIDWIFERY.

The matron of the Kensington Infirmary writes to correct a statement published in The Hospital, and signed "Nurse Edith," to the effect that those wishing free training should apply to the Kensington Infirmary. The matron says : "Be kind enough to have this notice corrected ; it is not the case at all, and the appearance of such a statement has given me much trouble. Training is given by us to one nurse, through the Workhouse Nursing Association; and to one other probationer, who pays a fee of £10, and remains with us three months in order to enable her to go in for the examination of the London Obstetrical Society, with a view of gaining a diploma for midwifery."

### A RIFT IN THE LUTE.

S. F. G. writes : "I am much interested in the British Nurses Association ; but, like most people, like to know who are the officials of any society I think of joining, and therefore wish to learn if there is any truth in the report that a difference, not to say dispute, has already arisen among the promoters of this Association ? Is it true that one of the ladies who originated the scheme and claims the secretaryship has had her right to the post disputed by a doctor, also one of the promoters, who claims it for himself ? I thought the special feature of the British Nurses Association was to be that it was managed entirely by nurses for nurses ; and if the orginators of the scheme don't mean to keep to that rule I don't see why they have separated themselves from the officials of the hospitals and nurse-training schools. I should really like to know the truth of the matter."

### THE BRITISH NURSES' ASSOCIATION.

In answer to a letter published in your paper on March 24th, I wish to know why a nurse who has worked hard for three years should not be registered on a footing with those that call themselves thoroughly-trained nurses, and boast of a certificate. I think the Society has a good guarantee when every nurse who joins the association must copy her certificate or testimonial on a form sent by Miss Wood ; so I do not think any nurse need be afraid, as the scheme stands, that any "Sairey Gamp" from a country village will be put on a footing with a nurse who holds a good certificate and testimonial certifying to three years' hospital training. If the writer, who signs herself "F. H. M.," refers back a few weeks to THE HOSPITAL, she will read the address given by Miss Wood to a body of nurses in St. George's Hall, at their first meeting; the writer will then think before she publishes letters to the public, which may prejudice nurses from joining the Association. I myself, as a certificated nurse and one who has joined the Association, heartily thank Miss Wood and the many ladies and gentlemen who have lent their names to the above, and am glad to see in the second opinion given in your paper that some nurses appreciate the scheme, and hope they will soon find the opportunity of becoming members.

London, April 3rd. A READER.

### Lectures.

APRIL 20th, at 1, Carlton Gardens, Pall Mall, at 3.30, Miss Manning on the Women of India. Admission 7s. 6d.

April 21st, at Wimbledon Drill Hall, at 7.30, Dr. Allinson on Rational Food and Feeding.

A course of four lectures on Opiates and Anæsthetics was given at Gresham College last week. The attendance was remarkably good, and several nurses in uniform were present each evening.

The forthcoming Gresham Lectures are on Astronomy, Law, Rhetoric, Geometry, and Music.

At Toynbee Hall a Physiology class for advanced students is now being held, and one on Practical Biology and Histology will shortly be commenced.

A course of University Extension Lectures on Experimental Chemistry will also be given in the same hall during the summer.

The lecture given at the Midwives Institute last week on the Nursing of Cases of Heart Disease contained many practical hints worth remembering. For instance, the lecturer advised nurses not to sprinkle turpentine on a cloth when it was to be applied for the alleviation of pain, but to put a tablespoonful of turpentine into a bowl of hot water, and wring the cloth out in that. The lecture was illustrated by diagrams and experiments, which added greatly to its interest.

### Anecdotes of Personal Adventure.

"I WAS once," said the Superintendent, "on the roof of the house, looking to some repairs, when two male patients, through the carelessness of the keepers, came up to me. One said, 'Doctor, here's a lark! We will all join hands and jump down!' 'That's not much to do, Mr. Jones,' I replied. 'Anybody can do that. Let us go down and then jump up; that's something worth doing.' They agreed, and we descended in the usual way.

ANOTHER time I was musing on the bank of our pond, where the water is deep. They came to me—(they had no business to be there)—and said, "Doctor, we know you're a famous swimmer." (I can't swim a stroke.) "We will pitch you in here." I saw they meant it. "To be sure," I said, "'tis a nice warm day for a swim; but this is a solemn occasion, and I propose that before my dip we sing together 'God Save the Queen.' They were charmed. I sang loud; they sang louder; we all roared and bellowed till a keeper heard us, and I returned to the House with a dry skin.

### Notes and Queries.

TO CORRESPONDENTS.—1. Questions may be written on post-cards. 2. Advertisements in disguise are inadmissible. 3. In answering a query please quote the number. 4. If a private answer is desired, a stamped addressed envelope must be forwarded.

(5) *Nursing Abroad.*—A trained nurse would like to know how to hear of any vacancy in hospital or district work abroad.

**Answers.**

(1) *Quotation Wanted*: It is from "The Sermon in the Hospital" in "The Disciples," by Eleanor Hamilton King. Published by Kegan Paul and Co., Paternoster Row.—A. E. C.

*Male Nurse* should apply to the Secretary of the Hospital named in his letter.

### Examination Questions.

THE following are the questions which were given by the Obstetrical Society at their examination for midwives on April 11th. About thirty-three candidates went up, quite two-thirds being nurses in uniform:—

(1.) You are called to a woman seven months pregnant bleeding copiously. What would you do?

(2.) Describe the mechanism of labour in a face presentation, the chin pointing to the right, and the occiput forward.

(3.) What are the chief points you would have to pay attention to in your first visit to a woman after her confinement? At what time would you visit her during the next fortnight?

(4.) Describe the appearance and character of the stool in new-born children: first, when healthy; second, when unhealthy. If unhealthy, how would you treat it?

The following are specimens of examination questions set to nursing probationers last summer:—

(1.) Describe what you would do to prevent the formation of a bed sore, and how would you treat one when existing?

(2.) To what points would you especially attend in the nursing of the following cases: compound fracture of the skull, excision of the tongue, tracheotomy, ovariotomy?

(3.) How would you give a hot-air bath? At what temperature should you give a cold bath, tepid bath, warm bath, hot bath—Fahrenheit scale?

(4.) State the methods you would adopt to ascertain the purity of milk. Describe the processes by which milk is digested.

(5.) Explain the following terms: Peritoneum, healing of a wound from the bottom, concussion of the brain, antiseptic dressing.

### Vacancies.

THE following vacancies are announced:—

**Matrons.**

National Orthopædic Hospital; £50. Southport Infirmary; £50.

**Nurses.**

Shrewsbury Infirmary; £25

Guildford Hospital; £12

Queen's Hospital, Birmingham.

Samaritan Free Hospital.

Dorset Hospital; two; £16 and £25.

Southport Infirmary; four; £20.

Bradford Infirmary; £25.

Liverpool Convalescent Institution; £20.

Blackburn Infirmary.

Nottingham Nursing Institution; £25.

Worthing Infirmary; £20.

Burton-on-Trent Infirmary; £25.

Convalescent Institution, Woolton, near Liverpool; £20.

Nurses' Home, Nottingham, Several; £25.

**District Nurses.**

Perth; for three months only. Glasgow Nursing Association.
Wantage, Berks.

**Probationers.**

Horton Infirmary, Banbury. Blackburn Infirmary.

**Midwives.**

Long Buckley, Rugby. Glasgow Nursing Association.

### Exchange and Mart.

UNDER this heading we propose to insert purely Exchange advertisements, at a charge of sixpence for eighteen words, or three insertions for one shilling. It must be distinctly understood that ordinary advertisements will not be inserted here.

### THE NATIONAL PENSION FUND FOR NURSES.

Incorporated February 17th, 1888. Established October 16th, 1887.

**PATRONESSES.**

THE COUNTESS OF ROSEBERY. LADY ROTHSCHILD.

**VICE-PRESIDENTS.**

THE EARL OF ABERDEEN. SIR EDMUND HAY CURRIE. E. A. HAMBRO, Esq.
LORD ROTHSCHILD. HENRY HUCKS GIBBS, Esq. JUNIUS S. MORGAN, Esq.

**THE COUNCIL.**

WALTER H. BURNS, Esq., *Chairman.*
HENRY C. BURDETT, Esq. (The Founder), *Deputy Chairman.*
J. S. BRISTOWE, Esq., M.D., F.R.S.
THOMAS BRYANT, Esq., F.R.C.S.
R. BRUDENELL CARTER, Esq., F.R.C.S.
F. C. CARR-GOMM, Esq.
PERCIVAL A. NAIRN, Esq.
EDWARD RAWLINGS, Esq.
MAJOR ROSS, M.P.
ALFRED CHARLES DE ROTHSCHILD, Esq.
J. C. STEELE, Esq., M.D.
JOHN WATNEY, Esq.
CLIFFORD WIGRAM, Esq.

**BANKERS.**—THE BANK OF ENGLAND AND ITS BRANCHES.

**CONSULTING ACTUARY.**—GEORGE KING, Esq., F.F.A., F.I.A. **SECRETARY.**—PHILIP GROVE, Esq.

*Prospectuses and all information may be obtained on application to the Secretary, 38, Old Jewry, London, E.C.*

